# Socio-economic inequalities in all-cause mortality during the COVID-19 period in north-western Tanzania, 2018–2021

**DOI:** 10.1186/s12963-025-00390-0

**Published:** 2025-07-14

**Authors:** Sophia Adam Kagoye, Charles Mangya, Eveline Konje, Jim Todd, Chodziwadziwa Kabudula, Jean Bashingwa, Jacqueline Materu, Coleman Kishamawe, Ties Boerma, Milly Marston, Mark Urassa

**Affiliations:** 1 Mwanza Research Centre, National Institute for Medical Research, Mwanza, Tanzania; 2 Department of Epidemiology and Biostatistics, Catholic University of Health and Allied Sciences, Mwanza, Tanzania; 3 Department of Population Health, London School of Hygiene and Tropical Medicine (LSHTM), London, UK; 4 MRC/Wits Rural Public Health and Health Transitions Research Unit (Agincourt), Faculty of Health Sciences, School of Public Health, University of the Witwatersrand, Johannesburg, South Africa; 5 Community Health Science, University of Manitoba, Winnipeg, Canada

## Abstract

**Background:**

Evidence suggests that the COVID-19 pandemic has exacerbated social and demographic inequalities in the communities through pathways of unequal exposure, vulnerability, and susceptibility. In Tanzania, evidence on COVID-19-related mortality is limited to health facility data, with little to no information on the mortality patterns in the general population. This study assessed sociodemographic inequalities in all-cause mortality during the COVID-19 period in north-western Tanzania.

**Methods:**

We utilized available longitudinal data from the Magu Health and Demographic Surveillance System (HDSS) from January 2018 to December 2021. We compared the crude death rates between subgroups of age, sex, area of residence, and wealth index for a period before (2018/2019) and during (2020/2021) the COVID-19 pandemic. To quantify how mortality risk varies across the subgroups we fitted a Cox proportional hazard model with an interaction of the COVID-19 period.

**Results:**

Overall mortality declined from 5.9 in 2018/2019 to 5.4 and 5.5 deaths per 1000 person-years in 2020 and 2021, respectively. We observed an increase in differences in crude death rates by age groups, area of residence, and wealth quintiles during the COVID-19 period. In the Cox proportional hazards model, compared to adults aged 15–49, we observed greater mortality risk in children under five (AHR:2.9; 95%CI: 2.2–3.9), older individuals aged 50–64 years (AHR:3.02; 95%CI:2.11–4.33) and 65 + (AHR:18.65; 95%CI:14.28–24.35) during COVID-19 period. Males were also at greater risk of death compared to females (AHR:1.30; 95%CI:1.06–1.59).

**Conclusion:**

Despite the overall mortality decline during the pandemic, we observed an increased risk of mortality among vulnerable subgroups (aged < 5 years and > 60 years) in the population. This highlights the need to take into account vulnerable subpopulations when addressing major public health issues in communities.

**Supplementary Information:**

The online version contains supplementary material available at 10.1186/s12963-025-00390-0.

## Background

COVID-19 has been described as a syndemic, an interaction of social and biological factors to produce poor health outcomes [1]. Low income and a reflection of social determinants of health such as poor housing, poor working conditions, unemployment, and poor healthcare access results in multiple, interacting, and additive risk factors for increased COVID-19 mortality through four inter-related pathways: unequal exposure, unequal transmission, unequal vulnerability, and unequal susceptibility [2, 3].

Evidence from high-income countries (HIC) suggests that the pandemic exacerbated social inequalities in mortality, with worse COVID-19-related outcomes among individuals from lower-income households and households experiencing housing insecurity [2, 4–6]. Globally, there are estimated 14.9 million excess deaths for 2020–2021 [7]. Currently, the WHO estimates about 7 million COVID-19 deaths, with the majority of the excess deaths (2.9 million) from the Americas, followed by Europe (2.2 million), Africa has reported the least number of excess deaths (175, 448) [8].

In Tanzania, the first case of COVID-19 was confirmed on the 16th of March 2020. The government quickly implemented several WHO-recommended preventive measures, such as public health information campaigns, closure of schools, banning large gatherings, restricting travel from affected countries, and quarantine of infected people [9, 10]. However, in May 2020 Tanzania relaxed the implementation of a lockdown to allow accessibility to health care services and citizens to continue working to meet their household’s needs [9, 10].

Information on COVID-19-related mortality in Tanzania is only limited to data from health facilities leaving a paucity of information on COVID-19-related mortality at a population level [11–13]. Quantitative synthesis to understand the magnitude of mortality and the degree of inequalities in all-cause mortality across different social stratifiers (age, socioeconomic position such as wealth, area of residence, and gender) at a population level during the COVID-19 pandemic is warranted. This is important to assist the authorities in better directing and aligning their response to future pandemics considering the importance of equity dimensions. In this study, we compare the socio-demographic inequalities in all-cause mortality before and during the COVID-19 pandemic at a population level in north-western Tanzania, using longitudinal data from the Magu health and demographic surveillance site (HDSS).

## Methods

### Study design

This study utilized available longitudinal data from Magu health and demographic surveillance sites from January 2018 to December 2021.

### Study setting

**Fig. 1:**
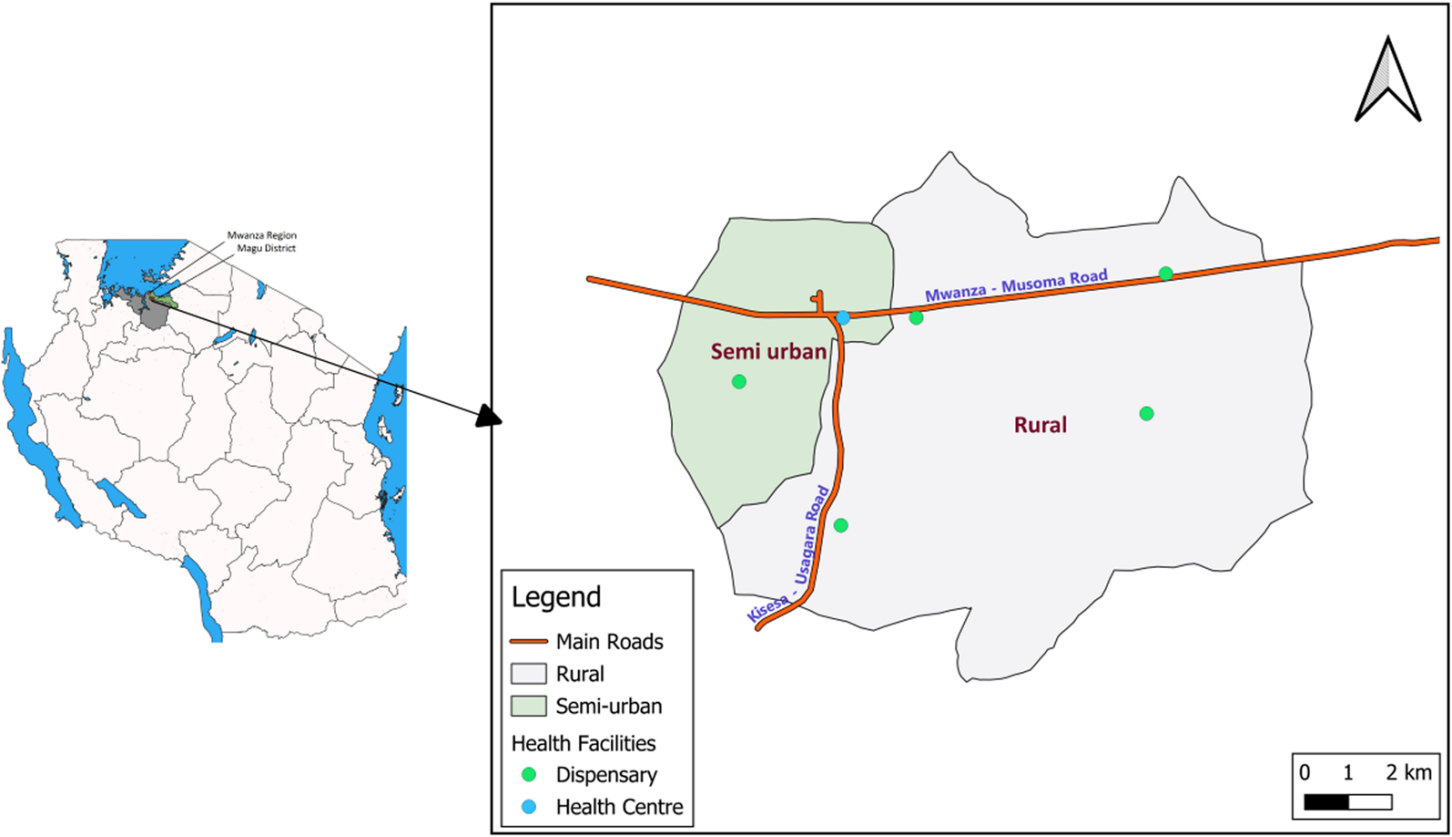
Map showing Semi-urban and rural areas in Magu Health and demographic surveillance site

Magu health and demographic surveillance system has been in operation since 1994 in a contiguous area of nine villages in the three wards in northwestern Tanzania (Kisesa, Bukandwe, and Bujora wards), 20 km east of the city of Mwanza, with a population of about 55,000 by 2022 (Fig. 1). The HDSS aims to measure child and adult mortality, fertility and mobility in the general population. The HDSS activities cover all the residents in the nine villages. Data collection began in 1994 with the baseline census, which involved listing of all individuals living the HDSS’s geographical area. Further details on Magu HDSS activities are explained elsewhere [14].

### Participants and study size

This study included all residents in the nine villages–four semi-urban villages and five rural villages–covered by Magu HDSS from a period of January 2018–December 2021. The study included a total of 163,319 total person-years of observations during the study period.

### Measurements

All individuals were listed with their names, sex, age, relation to the household head, education, source of income, household assets, material used in constructing the main family house, and survival status. All household residents were registered when first seen in the HDSS using a Demographic Household Card (DHHC) and are given a unique HDSS number. Demographic follow-up rounds have been constantly updated on a regular basis (at three, six- or twelve-monthly intervals) since the first census round in 1994. Information on household ownership of things for assessment of socioeconomic status within the site has been updated thrice since the baseline census, in 2004/05, 2018, and 2022 rounds. Socioeconomic status information used in this analysis was obtained from 2018.

#### Data collection during the COVID-19 pandemic

In common with the rest of Tanzania where activities continued during the pandemic, there was no interruption in collecting HDSS data during the pandemic. Research assistants were advised to observe social distancing while collecting data.

### Data management and Statistical analysis

Data were cleaned using Census and survey processing software (CS-Pro) and analyzed with Stata version 18.0 [15].

Using time-to-event analysis with calendar time as a time scale we obtained the total number of deaths and the person years of observation from all periods of observation (January 2018–December 2021), excluding any time a person migrated out of the cohort area. We further separated the periods based on the COVID-19 pandemic occurrence by splitting the data from 1st March 2020, specifying a period before (2018/2019) and the period during the COVID-19 pandemic (2020/2021) coded as 0 and 1 respectively.

We stratified all analyses by WHO age groups in years (< 5, 5–14, 15–49, 50–64, 65 +), sex (male, female), area of residence classified as semi-urban for individuals residing near the trading center (and nearer to the city), while the remaining being rural (Fig. 1) and household wealth quintiles calculated using principal component analysis from a list of household assets [16].

Crude death rates (CDR) were obtained and compared across the stratifiers for a period before and during the COVID-19 pandemic. Differences in CDR for a period before and during the COVID-19 pandemic between the most advantaged and the most disadvantaged subgroups of age (15–49/65 + years), sex (male/female), area of residence (urban/rural), and wealth quintiles (richest/poorest) were obtained as a measure of absolute inequalities. We presented the absolute changes between groups graphically using an equiplot [17], which makes it possible to visualize both the level of mortality in each group and the distance between groups. A larger distance between subgroups represents greater gaps and higher inequalities and a narrower distance represents narrower gaps and lower inequalities [17].

We further assessed how mortality risk varied across the sub groups of age, sex, area of residence, and wealth quintiles during the COVID-19 pandemic, by fitting a time-updated Cox proportional hazards model interacting COVID-19 period with all stratifiers in both Crude and Adjusted analyses. This allowed for a relaxation of the proportional hazard assumption by expanding the data on calendar time (i.e., time before and during COVID-19), as a result producing results on the calendar time update effect of covariates on all-cause mortality. We presented the measures of effect using the Hazards ratio with corresponding 95% CI, presenting the period before and during the COVID-19 pandemic, *p*-value of < 0.05 was considered statistically significant.

The overall goodness of fit of the model was assessed using Cox-Snell residuals, by plotting the cumulative hazard versus Cox-Snell residuals.

## Results

### Overall crude death rates

**Fig. 2:**
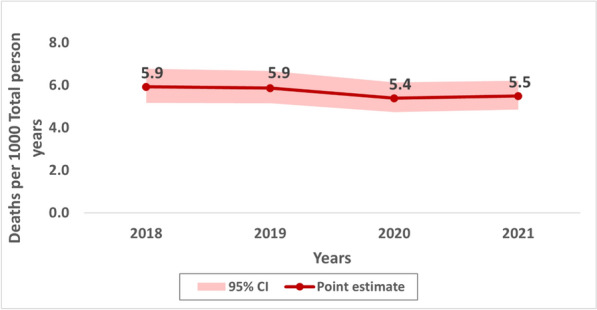
Overall crude death rates per 1000 with 95% CI in Magu Health and demographic surveillance site (2018–2021)

Overall, the crude death rate (CDR) per 1000 person-years (PY) for two years before the COVID-19 pandemic (2018/2019) remained unchanged at 5.9 deaths per 1000 PY then decreased to 5.4 in 2020 and slightly increased to 5.5 deaths per 1000 PY in 2021 (Fig. 2). Disaggregating by demographic and socio-economic stratifiers, in all four years higher number of deaths per 1000 PY were observed among older people aged 65 + , males, individuals residing in rural areas, and those from the poorest wealth quintile (Additional file 1).

### Absolute differences in CDR across sub-groups of age, sex, area of residence, and wealth quintiles, comparing the period before (2018/2019) and during (2020/2021) the COVID-19 pandemic

**Fig. 3:**
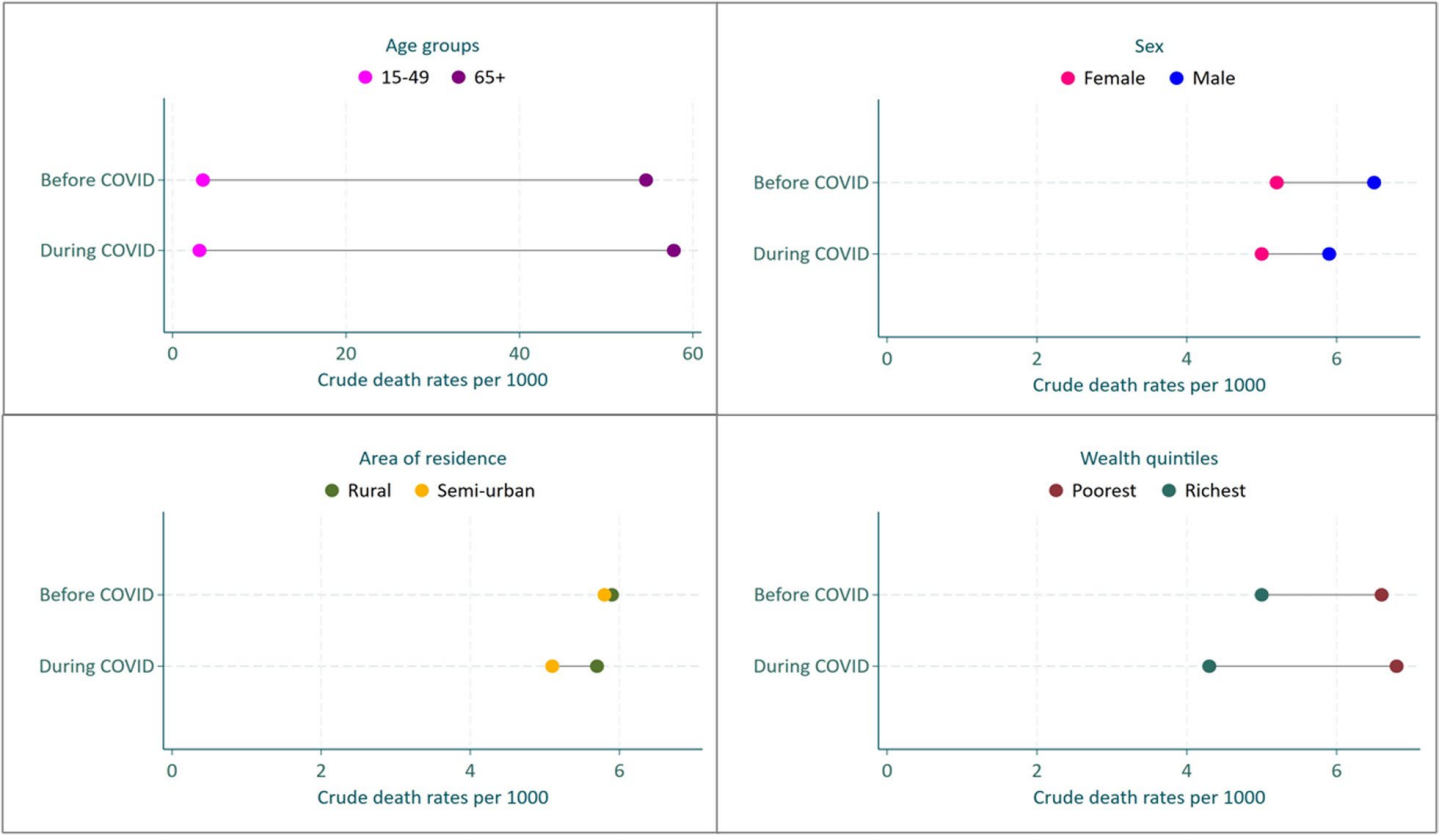
Equiplots showing absolute inequalities in CMR comparing before (2018/2019) and during (2020/2021) the COVID-19 pandemic

We compared CDR within the sociodemographic characteristics of age, sex, area of residence, and wealth quintile simply by subtracting CDR between the least advantaged and most advantaged subgroups. We presented the findings using equiplots presented in Fig. 3 and further details are in Additional file 1.

Differences in death rates by age groups (comparing 15–49-year-olds and older adults 65 + increased during the COVID-19 period (Fig. 3, Table S1). We observed an increase in CDR during COVID-19 among older adults aged 65 + from 54.6 (46.2, 64.5) to 57.8 (49.2, 67.9) deaths per 1000 TPY, while CDR among adults aged 15–49 years declined during the same period from 3.5 (2.9, 4.1) to 3.1 (2.5, 3.7) deaths per 1000 TPY.

The sex difference in CDR decreases from 1.3 difference before the COVID-19 period (2018/2019) to 0.9 difference during the COVID-19 period (2020/2021). CDR among both males and females were observed to decline with time (equiplots shifting to the left), and a greater decline was observed among males compared to the females (7.7% relative change among males compared to 1.9% relative change among females).

A similar pattern of mortality decline during the COVID-19 pandemic was observed when we compared rural/semi-urban residence, however, the absolute inequalities by the area of residence were observed to increase during the pandemic period (from a difference of 0.1 before to 0.6 during the pandemic).

The wealth gap in CDR was observed to increase during the COVID-19 pandemic from 1.6 to 2.5 absolute differences, with individuals from poor households having higher mortality compared to those from richest households in both periods.

### Effect of COVID-19 on mortality risk by background characteristics

The effect of COVID-19 on mortality risk across the subgroups of age, sex, area of residence, and wealth for a period before and during the COVID-19 pandemic after interacting each covariate with COVID-19 period is presented in Table 1.

**Table 1:** Effect of COVID-19 on mortality risk by background characteristics (Adjusted analysis)

	Before COVID-19 (2018/2019) ^∞^	During COVID-19 (2020/2021) ^€^
	AHR^¥^ (95% CI)	AHR^¥^ (95% CI)
*WHO age groups (Years)*		
< 5 5–14 15–49 50–64 65 +	2.49 (1.92, 3.23) *** 0.47 (0.33, 0.68) *** 1 2.97 (2.15, 4.09) *** 15.41 (12.02, 19.75) ***	2.94 (2.19, 3.94) *** 0.22 (0.13, 0.38) *** 1 3.02 (2.11, 4.33) *** 18.65 (14.28, 24.35) ***
*Sex*		
Male Female	1.36 (1.13, 1.64) ** 1	1.30 (1.06, 1.59) * 1
*Area of residence*		
Semi-urban Rural	1 0.83 (0.66, 1.03)	1 1.11 (0.87, 1.43)
*Wealth quintiles*		
Poorest Poorer Middle Richer Richest	1.44 (1.01, 2.05) * 1.30 (0.94, 1.79) 1.24 (0.89, 1.73) 0.97 (0.69, 1.37) 1	1.41 (0.95, 2.11) 1.01 (0.69, 1.48) 1.31 (0.90, 1.92) 1.33 (0.91, 1.93) 1

^*^p < 0.05; **p < 0.01; ***p < 0.001; ^¥^Adjusted Hazards ratio; ^**∞**^Interaction: covid0#covariate; ^**€**^Interaction: covid1#covariate

During the COVID-19 period, after adjusting for other factors, we observed significant differences in mortality risks by age and sex. The differences in mortality risk by area of residence and wealth quintiles were not statistically significant.

Across the age groups, after adjusting for other factors, we observed a significantly higher risk of mortality among children under-five and elderly people aged 50–64 years and 65 + years compared to adults aged 15–49 years before and during the COVID-19 period. During the COVID-19 period, the mortality risk ratio was more than twice among children under five (AHR: 2.94; 95% CI: 2.19, 3.94), it was three times higher among elder people aged 50–64 years (AHR: 3.02; 95% CI: 2.11, 4.33) and was 18 times higher among elder people 65 + (AHR: 18.65; 95% CI: 14.28, 24.35) compared to adults aged 15–49 years (Table 1).

Comparing males to females, in an adjusted analysis, we observed a significantly higher mortality risk among the males compared to females in both periods; where the increased mortality risk was 36% before the pandemic (AHR:1.36; 95% CI: 1.13, 1.64) declining to 30% higher during the pandemic (AHR: 1.30; 95% CI: 1.06, 1.59).

The mortality risk comparisons by socio-economic status were not statistically significant although compared to the Richest quintile, mortality risk among individuals in the poorest quintile was observed to be higher across both periods: By 44% before the COVID-19 pandemic (AHR: 1.44; 95% CI: 1.01, 2.05) and by 41% during the pandemic (AHR: 1.41; 95% CI: 0.95, 2.11).

## Discussion

This study examined socio-demographic disparities in all-cause mortality during the COVID-19 pandemic using population data from Magu HDSS in north-western Tanzania. Overall, we observed a decrease in mortality during the pandemic, indicating that COVID-19 did not significantly impact overall mortality in our study area. Similar trends were noted in other HDSS sites across Sub-Saharan Africa (SSA), including Gambia and rural Kenya [18], contrasting with observations in Western countries [19]. In Tanzania, most COVID-19 mortality studies have been hospital-based cross-sectional analyses, revealing mortality rates of 31–34% in tertiary hospitals [11–13]. While additional qualitative research would provide valuable contextual insights into these trends, such investigation was beyond the scope of our study.

When analyzing mortality risk by age group during the pandemic compared to pre-pandemic periods, we found higher risk ratios for mortality among children under five and the elderly (aged 50–64 and 65 + years) compared to adults aged 15–49 years. This pattern mirrors findings in both high-income settings and SSA, including Tanzania as reported in health facilities [11, 13, 19–21]. Elevated mortality risk among the elderly suggests a direct pandemic impact, potentially linked to lower immunity and higher prevalence of non-communicable diseases, known COVID-19 risk factors [11, 20]. Hospital studies in Tanzania have also indicated higher case-fatality rates among older adults with comorbidities like diabetes and hypertension compared to younger age groups [11, 12]. Meanwhile, increased mortality risk among children under five might be attributed to disruptions in maternal and child health services during the pandemic, prioritizing COVID-19 care over routine services [22].

Our study identified significantly higher mortality risk among males compared to females, with a narrowing of the gender gap during the COVID-19 period. This aligns with what is observed in other studies [20, 19, 18], the differences in COVID-19 mortality risks among males and females are likely attributed to sex-based biological, social, and behavioral factors related to gender [18]. The slower decline in female mortality during the pandemic in our study area suggests potential disparities in health service access or increased maternal health issues during the pandemic. Further investigation into service utilization and major causes of death among males and females during the pandemic is warranted.

While not statistically significant, our findings suggest an increase in absolute inequalities by wealth quintiles, with higher mortality risks observed among individuals from poorer households compared to wealthier counterparts. This socioeconomic disparity, likely exacerbated by overcrowded living conditions and higher non-communicable disease burdens among the socially disadvantaged population within the society, is consistent with observations in other settings [20].

Using longitudinal population-based data from a contiguous geographic area provided valuable insights into mortality inequalities during the COVID-19 pandemic at a population level in Tanzania, where such information is limited. However, the study's limitation includes its focus on a subnational area, possibly under-representing urban dynamics where COVID-19 exposure might differ.

## Conclusion

In conclusion, while the overall impact of COVID-19 on all-cause mortality in our study area was minimal, disparities by age, sex, and socioeconomic status were pronounced, demonstrating that the impact of COVID-19 within sub-Saharan African communities may not be directly comparable to the rest of the world, or even to each other. Future public health responses should consider these indirect effects on vulnerable groups and address equity dimensions in emergency planning and community interventions.

## Supplementary Information

Supplementary material 1

## Data Availability

Data for computing mortality indicators by year, age, and sex are available from the MRC/Wits Agincourt Research Unit Data Repository (https://data.agincourt.co.za/index.php/catalog/346). Data containing other covariates used in the analysis reported in this manuscript can be accessed through a formal request to the corresponding author.

## Abbreviations

CDR: Crude death rates
HDSS: Health and demographic surveillance site
PY: Person years
