# Supplementary material for: Socio-economic inequalities in all-cause mortality during the COVID-19 period in north-western Tanzania, 2018–2021
Source: Popul Health Metr. 2025 Jul 14;23(Suppl 2):39. doi: 10.1186/s12963-025-00390-0 (PMC12261527; doi:10.1186/s12963-025-00390-0)
Supplement: Supplementary file 1 — Supplementary material 1 [file 12963_2025_390_MOESM1_ESM.docx]

**Additional file 1**

**Impact of COVID-19 on socio-demographic inequalities in all-cause mortality in north-western Tanzania, 2018-2021**

**Supplementary Materials**

**Table S1: Total number of deaths per 1000 person-years of observation (2018-2021)**

| **Characteristic** | **2018** | | **2019** | | **2020** | | **2021** | |
| --- | --- | --- | --- | --- | --- | --- | --- | --- |
|  | **Deaths** | **Deaths per 1000 PY^*^** | **Deaths** | **Deaths per 1000 PY^*^** | **Deaths** | **Deaths per 1000 PY^*^** | **Deaths** | **Deaths per 1000 PY^*^** |
| **Overall** | 210 | 5.9 | 230 | 5.9 | 228 | 5.4 | 254 | 5.5 |
| **WHO age groups (Years)**  <5  5-14  15-49  50-64  65+ | 41  17  55  28  69 | 7.1  1.5  3.6  11.8  62.2 | 63  23  63  22  59 | 9.9  1.9  3.7  8.6  48.4 | 65  11  56  30  66 | 9.9  0.8  3.0  10.9  49.1 | 73  9  58  22  92 | 10.0  0.6  2.8  7.5  64.9 |
| **Sex**  Male  Female | 109  101 | 6.3  5.6 | 125  105 | 6.5  5.2 | 126  102 | 6.1  4.7 | 134  120 | 6.0  5.0 |
| **Residence**  Semi-urban  Rural | 91  119 | 5.8  6.0 | 105  125 | 5.8  5.9 | 92  136 | 4.8  5.9 | 114  140 | 5.5  5.5 |
| **Wealth quintiles** |  |  |  |  |  |  |  |  |
| Poorest | 50 | 6.4 | 53 | 7.0 | 53 | 7.0 | 48 | 6.3 |
| Poorer | 50 | 6.4 | 62 | 6.0 | 50 | 4.9 | 49 | 5.0 |
| Middle | 38 | 5.5 | 45 | 6.7 | 47 | 6.9 | 35 | 5.2 |
| Wealthier | 31 | 4.8 | 28 | 4.4 | 35 | 5.7 | 39 | 6.6 |
| Wealthiest | 33 | 5.1 | 33 | 5.1 | 23 | 3.7 | 29 | 4. |
| **person-years* | | | | | | | | |

**Table S2: Crude mortality rate and absolute inequalities in CMR across subgroups comparing the period before (2018/2019) and during COVID-19 (2020/2021) pandemic**

| **Characteristics** | **Number of deaths** | | **Mortality per 1000 PY^*^ (95% CI)** | | **Absolute differences in CDR^¥^** | |
| --- | --- | --- | --- | --- | --- | --- |
|  | **Before COVID-19 (2018/2019)** | **During COVID-19**  **(2020/2021)** | **Before COVID-19 (2018/2019)** | **During COVID-19 (2020/2021)** | **Before COVID-19 (2018/2019)** | **During COVID-19**  **(2020/2021)** |
| **Overall** | 441 | 447 | 5.8 (5.3, 6.4) | 5.5 (5.0, 6.1) |  |  |
| **WHO age groups (Years)** |  |  |  |  |  |  |
| <5 | 119 | 123 | 9.0 (7.6, 10.8) | 9.6 (8.0, 11.4) |  |  |
| 5-14 | 42 | 18 | 1.7 (1.2, 2.2) | 0.7 (0.5, 1.1) |  |  |
| 15-49 | 122 | 110 | 3.5 (2.9, 4.1) | 3.1 (2.5, 3.7) |  |  |
| 50-64 | 54 | 48 | 10.1 (7.7, 13.2) | 9.1 (6.7, 12.1) |  |  |
| 65+ | 139 | 147 | 54.6 (46.2, 64.5) | 57.8 (49.2, 67.9) | 51.1 | 54.7 |
| **Sex** |  |  |  |  |  |  |
| Male | 258 | 236 | 6.5 (5.7, 7.3) | 5.9 (5.3, 6.9) | 1.3 | 0.9 |
| Female | 218 | 210 | 5.2 (4.6, 6.0) | 5.0 (4.4, 5.8) |  |  |
| **Area of residence** |  |  |  |  |  |  |
| Semi-urban | 213 | 189 | 5.8 (5.0, 6.6) | 5.1 (4.4, 5.9) |  |  |
| Rural | 263 | 257 | 5.9 (5.2, 6.7) | 5.7 (5.1, 6.5) | 0.1 | 0.6 |
| **Wealth quintiles** |  |  |  |  |  |  |
| Poorest | 109 | 95 | 6.6 (5.4, 7.9) | 6.8 (5.6, 8.3) | 1.6 | 2.5 |
| Poorer | 124 | 87 | 6.3 (5.2, 7.5) | 4.7 (3.8, 5.9) |  |  |
| Middle | 88 | 77 | 6.0 (4.8, 7.4) | 6.2 (5.0, 8.0) |  |  |
| Richer | 67 | 66 | 4.9 (3.8, 6.2) | 6.0 (4.7, 7.6) |  |  |
| Richest | 69 | 49 | 5.0 (3.9, 6.3) | 4.3 (3.3, 5.7) |  |  |
| **Person-years;* **^¥^**Age (65 – 15-49), Sex (Male-Female), Area of residence (Rural – Semi-urban), Wealth quintiles (Poorest-Richest) | | | | | | |

**Table S3: Mortality risk by background characteristics before and during COVID-19 periods (Crude & Adjusted analysis)**

| **Characteristic** | **Before COVID-19** | | **During COVID-19** | |
| --- | --- | --- | --- | --- |
|  | **Crude analysis*^∞^*** | **Adjusted analysis*^∞^*** | **Crude analysis^€^** | **Adjusted analysis^€^** |
|  | **CHR (95% CI) ^β^** | **AHR (95% CI)** **^¥^** | **CHR (95% CI) ^β^** | **AHR (95% CI) ^¥^** |
| **WHO age groups (Years)** |  |  |  |  |
| <5 | 2.62 (2.03, 3.37) *** | 2.49 (1.92, 3.23) *** | 3.14 (2.43, 4.06) *** | 2.94 (2.19, 3.94) *** |
| 5-14 | 0.48 (0.34, 0.69) *** | 0.47 (0.33, 0.68) *** | 0.24 (0.14, 0.39) *** | 0.22 (0.13, 0.38) *** |
| 15-49 | 1 | 1 | 1 | 1 |
| 50-64 | 2.93 (2.13, 4.04) *** | 2.97 (2.15, 4.09) *** | 3.01 (2.15, 4.23) *** | 3.02 (2.11, 4.33) *** |
| 65+ | 15.77 (12.37, 20.12) *** | 15.41 (12.02, 19.75) *** | 19.06 (14.88, 24.40) *** | 18.65 (14.28, 24.35) *** |
| **Sex** |  |  |  |  |
| Male | 1.24 (1.04, 1.49) * | 1.36 (1.13, 1.64) ** | 1.19 (0.99, 1.43) | 1.30 (1.06, 1.59) * |
| Female | 1 | 1 | 1 | 1 |
| **Area of residence** |  |  |  |  |
| Semi-urban | 1 | 1 | 1 | 1 |
| Rural | 1.03 (0.86, 1.24) | 0.83 (0.66, 1.03) | 1.12 (0.93, 1.36) | 1.11 (0.87, 1.43) |
| **Wealth quintiles** |  |  |  |  |
| Poorest | 1.33 (0.98, 1.79) | 1.44 (1.01, 2.05) * | 1.58 (1.08, 2.15) ** | 1.42 (0.95, 2.11) |
| Poorer | 1.25 (0.93, 1.68) | 1.30 (0.94, 1.79) | 1.09 (0.77, 1.55) | 1.01 (0.69, 1.48) |
| Middle | 1.21 (0.88, 1.65) | 1.24 (0.89, 1.73) | 1.43 (1.00, 2.05) * | 1.31 (0.90, 1.92) |
| Richer | 0.98 (0.70, 1.37) | 0.97 (0.69, 1.37) | 1.38 (0.95, 1.99) | 1.33 (0.91, 1.93) |
| Richest | 1 | 1 | 1 | 1 |

**p<0.05; **p<0.01; ***p<0.001;* **^β^** Crude Hazards ratio; **^¥^**Adjusted Hazards ratio; **^∞^**Interaction: covid0#covariate; **^€^**Interaction: covid1#covariate
